# The impact of pharmaceutical care on the clinical outcome of diabetes mellitus among a rural patient population

**DOI:** 10.4103/0973-3930.41981

**Published:** 2008

**Authors:** K. P. Arun, R. Murugan, M. Rajesh Kanna, S. Rajalakshmi, R. Kalaiselvi, V. Komathi

**Affiliations:** Department of Pharmacy Practice, Kamalakshi Pandurangan College of Pharmacy, Tiruvannamalai - 606 603, Tamil Nadu, India

**Keywords:** Diabetes mellitus, health-related quality of life, Indian population, patient counseling, pharmaceutical care

## Abstract

**AIM:**

To evaluate the impact of pharmaceutical care on the clinical outcomes of patients enrolled in a pharmacist-coordinated diabetes management program in a rural health setup.

**SETTINGS AND DESIGN:**

Patients were registered into ‘control’ and ‘intervention’ groups by randomization at three primary health centers. The study was an open-label parallel study.

**MATERIALS AND METHODS:**

Medical records were prospectively reviewed. Capillary blood glucose level, blood pressure and demographic data were collected at baseline and at the follow-up visits. Pharmacists gave counseling to the intervention group during every visit and their health-related quality of life (HRQoL) was assessed with the Ferrans and Powers questionnaire.

**STATISTICAL ANALYSIS:**

Single factor ANOVA and the t-test were used to compare the results using SPSS version 0.9 software and MS Excel worksheets.

**RESULTS:**

The intervention group (*n* = 104) showed well-controlled BMI, whereas the control group (*n* = 50) showed significant increase in the BMI. Mean blood glucose level in the intervention group reduced to 25 units from baseline (*P* = 0.0001) but was significantly increased in the control group (*P* = 0.0001). ANOVA showed that from the second follow-up onward there was significant decrease in blood glucose levels. Overall, the HRQoL scores increased by 45% in the intervention group and decreased by 2% in the control group.

**CONCLUSIONS:**

The pharmaceutical care program was effective in improving the clinical outcome and HRQoL of diabetes patients in rural India. Such ‘pharmaceutical care’ models should be fine-tuned and implemented widely.

## Introduction

Many pharmaceutical care programs have been established in various countries to enhance clinical outcomes and the health-related quality of life (HRQoL). These programs were implemented by pharmacists, with the cooperation of the physicians and other health care professionals.[1–3] However, such programs are not very common in the Indian scenario. The community pharmacist is a professional with knowledge of medicines and health care and is easily accessible to patients throughout the day. The pharmacist can, therefore, in collaboration with doctors and other health care professionals, contribute to the improvement of diabetic patients' quality of life by informing and educating patients, answering their questions and, at the same time, monitoring the treatment they receive and carrying out their own assessments of patients' health.[4] India is becoming the diabetes capital of the world, with an estimated 30 million diabetics today[5] and the numbers set to increase to 73 million by 2025.[6] With good glycemic control, several long-term, life-threatening complications of diabetes can be prevented.[7] The community pharmacist's involvement in diabetes care is justified by his or her position as a key member of the health care team; the need is to work together with other health professionals to prevent diabetes and its complications.[4] The main aim of this work is to evaluate the impact of ‘pharmaceutical care’ provided to outpatients with diabetes mellitus (DM) and thereby to generate external clinical evidence in the Indian context.

## Materials and Methods

This 6-month, prospective, open-label study was conducted with type 2 diabetic patients from three primary health centers (PHCs) of a district in Northern Tamil Nadu after getting ethical clearance. Adult patients aged over 18 years and taking medications for type 2 DM for at least the past 12 months were included. Pregnant women were excluded from the study. After getting written consent from the patients they were allocated into ‘control’ and ‘intervention’ groups by 1:2 randomization. The questionnaire for collecting the demographic data of the patients and for assessing health-related quality of life (HRQoL) was prepared with reference to Ferrans and Powers Quality of Life Index-Diabetes version III.[8] This questionnaire was translated into Tamil and distributed to the patients. A Pulsatum - Digital biosensor blood glucose monitor was validated and used to measure the capillary blood glucose level. Blood pressure (BP), body weight, and height were recorded with standard instruments.

All the baseline information and demographic data were collected in the prescribed format by direct interaction with the patients of both groups. To avoid bias, a lab technician at the respective PHC interviewed the patients and assisted them in filling up the HRQoL forms. Then the capillary blood glucose level (fasting) was measured and documented; BP was also checked. Following this, the intervention group patients were given counseling about diabetes and health care; the control group received no counseling. The intervention group was followed up every month (for 5 months). At each visit, capillary blood glucose and BP were measured and documented and brief counseling was given. At the end of study the HRQoL form was filled up again for both the groups of patients in a similar fashion. At the end of the study the capillary blood glucose and BP were measured for the patients of the control group and documented. Then the same counseling that was given to intervention group of patients was also given to the control group. All the data were analyzed using the computer software SPSS version 0.9 and MS Excel. The HRQoL data were analyzed using the Excel program of Ferrans and Powers.

## Results

A total of 104 patients in the intervention group and 50 patients in the control group completed the study. The demographic data like sex, age, body weight, height, body mass index (BMI), marital status, education, occupation, disease duration and habits of smoking and alcohol at baseline are comparable between the two groups [Table 1]. The mean body weight of the intervention group at baseline was 55.75 ± 8.61 kg, which was reduced to 54.28 ± 8.12 kg at the end of the study; this difference is highly significant (*P* = 0.0001). The BMI profile showed that the patients in the intervention group have well-controlled BMI, whereas the control group showed considerable increase in the BMI. At the end of the study, the mean capillary blood glucose level in the intervention group reduced to about 25 mg/dl from the baseline value, which was highly significant (*P* = 0.0001). In the control group, the mean capillary blood glucose level was increased to about 15 mg/dl from the baseline, which is also extremely significant (*P* = 0.0001). The mean values of systolic and diastolic BP were significantly increased from the baseline values in the control group. In the intervention group, a decrease of about 9 units from the baseline in the mean value of systolic BP and an increase of 2 mm Hg in the diastolic pressure was observed. Here, the increase in the diastolic value may be due to the combined effect of nonhypertensive diabetic patients and hypertensive diabetic patients. The mean capillary blood glucose levels of the intervention group in five consecutive follow-ups were treated for analysis of variance (ANOVA - single factor) with baseline values (*F* value = 3.84); this showed that there is no significant difference between the first follow-up and baseline (*F* = 0.47). But follow-ups 2, 3, 4 and 5 months showed significant differences (*F* = 14.24, 24.70, 22.49 and 37.63, respectively) [Table 2].

**Table 1:** Baseline data

Parameter	Control group	Intervention group
*n*	50	104
Sex (*n*)
Male	24	46
Female	26	58
Age (years)*	58.8 ± 9.95	57.5 ± 8.63
Weight (kg)*	55.8 ± 7.62	55.75 ± 8.61
BMI (kg/m^2^)*	21.14 ± 2.89	21.15 ± 3.01
Fasting plasma glucose level (mg/dl)*	134.38 ± 20.46	140.08 ± 24.16
Blood pressure (mm Hg)*
Systolic	126.56 ± 10.15	134.79 ± 16.03
Diastolic	77.92 ± 9.89	78.5 ± 9.19

* Values are in mean ± SD

**Table 2:** Changes in the parameters from baseline to the endpoint

Parameter	Control group (*n* = 50) (mean ± SD)	Intervention group (*n* = 104) (mean ± SD)	*P*
Change in body weight (kg)	1.14 ± 0.78	−1.46 ± 1.07	0.0001*
Change in BMI (kg/m^2^)	0.43 ± 0.29	−0.55 ± 0.40	0.0001*
Change in fasting plasma glucose level (mg/dl)	14.9 ± 11.24	−24.94 ± 12.54	0.0001*
Blood pressure (mm Hg)
Systolic	6.64 ± 14.22	−9.13 ± 13.67	0.0001*
Diastolic	2.84 ± 9.16	2.25 ± 8.82	0.7050†

* Highly significant

† not significant

**Figure 1:**
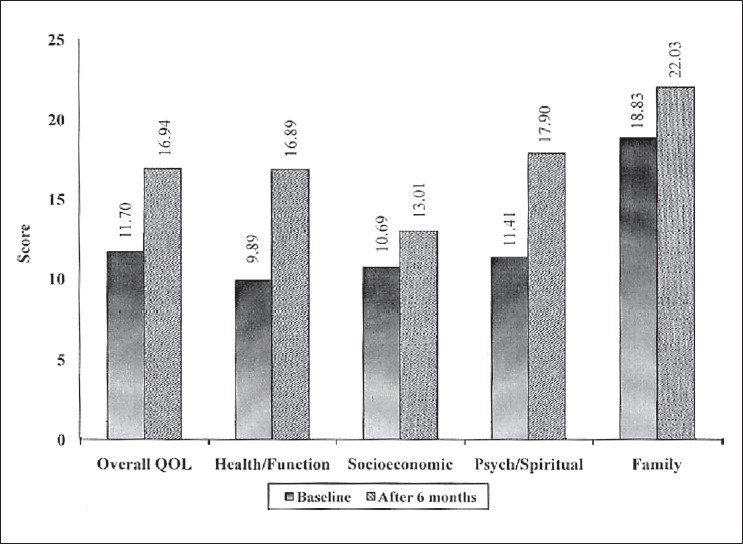
Scores in the different domains of quality of life among the intervention group patients at baseline and at the end of study

**Figure 2:**
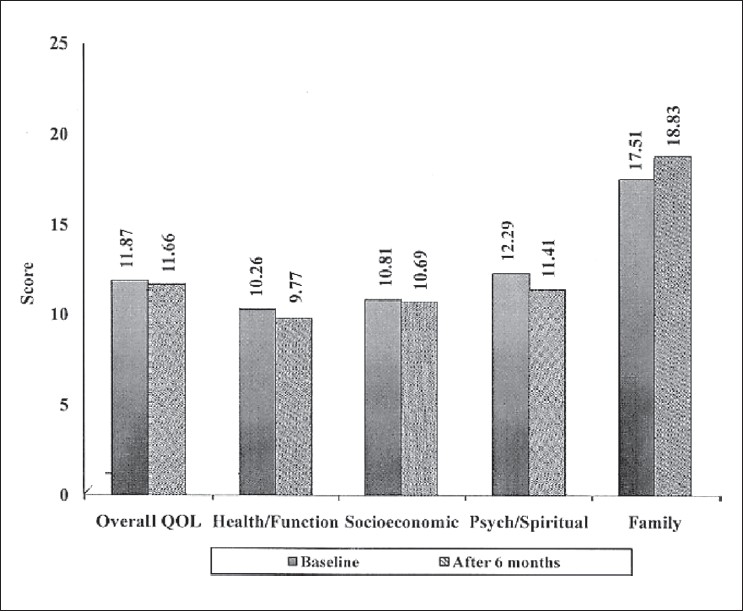
Scores in the different domains of quality of life among the control group patients at baseline and at the end of study

The overall HRQoL score was increased by about 45% from the baseline in the intervention group. Individual areas like the health/function, socioeconomic, psychological/spiritual and family domains show positive changes, ranging from 17-71% [Figure 1]. In contrast, the overall HRQoL score of the control group was decreased by about 2% and except the family domain all other domains show negative changes [Figure 2].

## Discussion

The pharmacist's involvement in diabetes care is justified by his or her position as a key member of the health care team; he or she needs to work together with other health professionals to prevent diabetes and its complications.[4] It is assumed that such pharmacist-involved diabetic care programs cannot actually be conducted in practice, but this should be refuted and the benefit should go to the rural patients who form the majority in developing countries like India. This study has shown that pharmaceutical care will help to improve glycemic control and other vital parameters like BMI and, more importantly, such professional care will help to improve the overall HRQoL of diabetic patients. The results are comparable with that of similar studies conducted in the Western world.[1,2] The 6-month study duration is long enough to assess the changes in the parameters. However, we could not estimate the glycosylated hemoglobin in this study as was done in other studies conducted in developed countries. Even today, in India, the common clinical practice when monitoring diabetes mellitus is to measure fasting blood glucose. Therefore we should not underestimate the value of such investigations. Thus, as an experimental attempt, we provided pharmaceutical care to a small group of patients and proved that such services can add value to the health care provided. With the wealth of graduate pharmacists in developing countries like India, such ‘pharmaceutical care’ models should be fine-tuned and implemented, which will be another milestone for the profession of pharmacy.
